# Effects of reducing sedentary behaviour on renal glucose uptake during insulin stimulation: A post‐hoc analysis of a 6‐month randomized controlled trial

**DOI:** 10.1111/dom.16631

**Published:** 2025-07-22

**Authors:** Eleni Rebelos, Prince Dadson, Tanja Sjöros, Saara Laine, Jooa Norha, Taru Garthwaite, Eliisa Löyttyniemi, Olli Eskola, Mikko Koivumäki, Henri Vähä‐Ypyä, Harri Sievänen, Tommi Vasankari, Jussi Hirvonen, Kirsi Laitinen, Noora Houttu, Kari K. Kalliokoski, Juhani Knuuti, Ele Ferrannini, Andrea Mari, Ilkka Heinonen

**Affiliations:** ^1^ Turku PET Centre University of Turku, Åbo Akademi University and Turku University Hospital Turku Finland; ^2^ Department of Clinical and Experimental Medicine University of Pisa Pisa Italy; ^3^ Department of Biostatistics University of Turku and Turku University Hospital Turku Finland; ^4^ The UKK Institute for Health Promotion Research Tampere Finland; ^5^ Faculty of Medicine and Health Technology, Tampere University Tampere Finland; ^6^ Department of Radiology University of Turku and Turku University Hospital Turku Finland; ^7^ Institute of Biomedicine and Nutrition and Food Research Center, University of Turku Turku Finland; ^8^ Department of Biomedical Engineering Huazhong University of Science and Technology Wuhan Hubei China; ^9^ CNR Institute of Clinical Physiology Pisa Italy; ^10^ CNR Institute of Neuroscience Padua Italy

**Keywords:** clinical physiology, clinical trial, exercise intervention, insulin resistance, randomised trial

## Abstract

**Aims:**

Obesity is an independent risk factor for chronic kidney disease, and weight loss interventions lead to better kidney outcomes. We aimed to assess whether reducing sedentary behaviour in patients with metabolic syndrome impacts renal glucose uptake rate (GU) during insulin stimulation.

**Materials and Methods:**

Forty‐four participants with metabolic syndrome were randomized to receive either guidance to reduce sedentary behaviour (INT) by 1 h/day during a 6‐month intervention or to maintain usual sedentary behaviour (CONT). For this post‐hoc analysis, we included all participants with available renal data: 34 participants at baseline and 30 at the end of the intervention. Participants underwent ^18^F‐fluorodeoxyglucose positron emission tomography ([^18^F]FDG‐PET) during a hyperinsulinemic clamp at baseline and at 6 months. Renal [^18^F]FDG‐PET data were analysed using fractional uptake rate (FUR). A correction for the estimated residual amount of [^18^F]FDG inside the tubuli was applied. Corrected GU was calculated as the product of FUR and glycemia.

**Results:**

At the study end, light and moderate‐to‐vigorous physical activity (PA) were increased and BMI was slightly decreased, with no significant intervention effect. Cortical and medullary GU increased vs baseline, similarly in both groups. At baseline, cortical GU was directly related to the degree of insulin sensitivity and inversely to BMI and circulating FFA levels. Change in renal GU was directly related to change in liver GU, but not to the change in whole‐body insulin sensitivity.

**Conclusions:**

In patients with metabolic syndrome, insulin‐stimulated renal GU increases concomitantly with a small decrease in body adiposity, independently of changes in whole‐body glucose disposal.

## 1 INTRODUCTION

Sedentary behaviour is defined as activities that involve energy expenditure below 1.5 metabolic equivalents, such as sitting, lying down, and watching television.^1^ Increasing evidence suggests that time spent in sedentary behaviour is associated with increased adiposity,^2^ metabolic syndrome,^3^ abnormal glucose regulation,^4^ and mortality,^5^ regardless of moderate‐to‐vigorous‐intensity physical activity level. Moreover, studies have shown that sitting for long periods is linked to an increased risk of kidney function decline, which remains significant even when accounting for confounding factors such as existing chronic kidney disease (CKD) risk and background physical activity level.^6^, ^7^

Previous data suggest that physical activity, independent of weight loss, improves skeletal muscle insulin sensitivity, mediated by accelerated translocation of GLUT4 transporters to the plasma membrane and increased glucose transport.^8^ Whether increasing physical activity (or reducing sedentary time) improves renal tissue‐specific glucose uptake has thus far not been addressed. Investigating this could have important implications for understanding how lifestyle changes affect kidney function.

Recently, our group has proposed a method whereby renal radioactivity from [^18^F]FDG‐PET scans may be used to assess renal glucose uptake.^9^, ^10^ The premise in these studies was a sufficiently late kidney scan after [^18^F]FDG injection, and a meticulous documentation of the last urination before the scan, urine volume, and [^18^F]FDG excreted in the urine. Using this approach, we have shown that the renal cortex acts as an insulin sensitive tissue in humans. In this post hoc analysis we used data from a randomised controlled trial in which the primary outcome was the change in insulin sensitivity following a daily 1‐h reduction in sedentary behaviour.^11^ Our aim was to assess whether a 6‐month intervention aiming to reduce sedentary behaviour by 1 h per day improves glucose uptake under insulinised conditions in the renal cortex and medulla of previously sedentary, inactive adults with metabolic syndrome.

## 2 MATERIALS AND METHODS

### 2.1 Study design and study participants

This trial aimed to assess whether a daily reduction of sedentary behaviour by 1 h has an effect on insulin sensitivity. The study consisted of a 4‐week screening phase and a 6‐month intervention period and was conducted at the Turku PET Centre, Turku, Finland, from April 2017 to March 2020 in accordance with good clinical practice guidelines and the Declaration of Helsinki. Written informed consent was obtained from all participants prior to enrolment. Ethical approval was granted by the Ethics Committee of the Hospital District of Southwestern Finland (16/1801/2017), and the trial was registered at ClinicalTrials.gov (NCT03101228, registered on 05/04/2017).

Participants were allocated to intervention (INT) and control (CONT) groups by a statistician using random permuted blocks with a 1:1 allocation ratio. Randomization was performed separately for men and women. The details of the intervention have been previously described.^11^ Briefly, participants in the INT group were instructed to reduce their daily sedentary behaviour by 1 h compared to the baseline determined over a 4‐week screening period. This was supported by continuous accelerometry in combination with a mobile application (ExSed, UKK Terveyspalvelut Oy, Tampere, Finland), which allowed self‐monitoring of individual daily sedentary behaviour and physical activity goals. Participants received an individual counselling session at the beginning of the intervention, and strategies to achieve the goals were tailored to each participant's preferences. However, instead of intentional physical activity training per se, non‐exercise activities such as using standing desks, lightly walking while on the phone, and taking the stairs instead of an elevator were recommended. Participants in the CONT group were instructed to maintain their habitual physical activity and sedentary behaviour. All participants received 2–3 phone calls from a researcher and visited the research centre at least once during the intervention to receive support and ensure the proper functioning of the devices.

The inclusion and exclusion criteria of the study participants have been previously reported.^11^ Participants with overweight or obesity and metabolic syndrome, with age between 40 and 65 years, who were self‐reportedly physically inactive (less than 120 min per week of moderate‐to‐vigorous physical activity) and sedentary (at least 10 h per day or 60% of accelerometer wear time per day during screening) were recruited. Exclusion criteria included previous cardiac events, diagnosed diabetes, excessive alcohol consumption, use of narcotics or tobacco, depressive or bipolar disorder, and any chronic disease or condition that, according to the investigators, could compromise safety or the results of the study. Participants were not taking any medications known to affect insulin sensitivity. Among them, 7 women and 9 men had hypertension and received varying antihypertensive regimens: 3 participants were treated with an angiotensin‐converting enzyme inhibitor (ACEi) or angiotensin II receptor blocker (ARB); 3 with ACEi/ARB combined with a dihydropyridine calcium channel blocker (CCB); 6 with an ACEi/ARB and a diuretic; 1 with a beta‐blocker; 1 with ACEi/ARB, diuretic and beta‐blocker; 1 with a dihydropyridine CCB and beta‐blocker; and 1 with ACEi/ARB, diuretic and dihydropyridine CCB.

**FIGURE 1:**
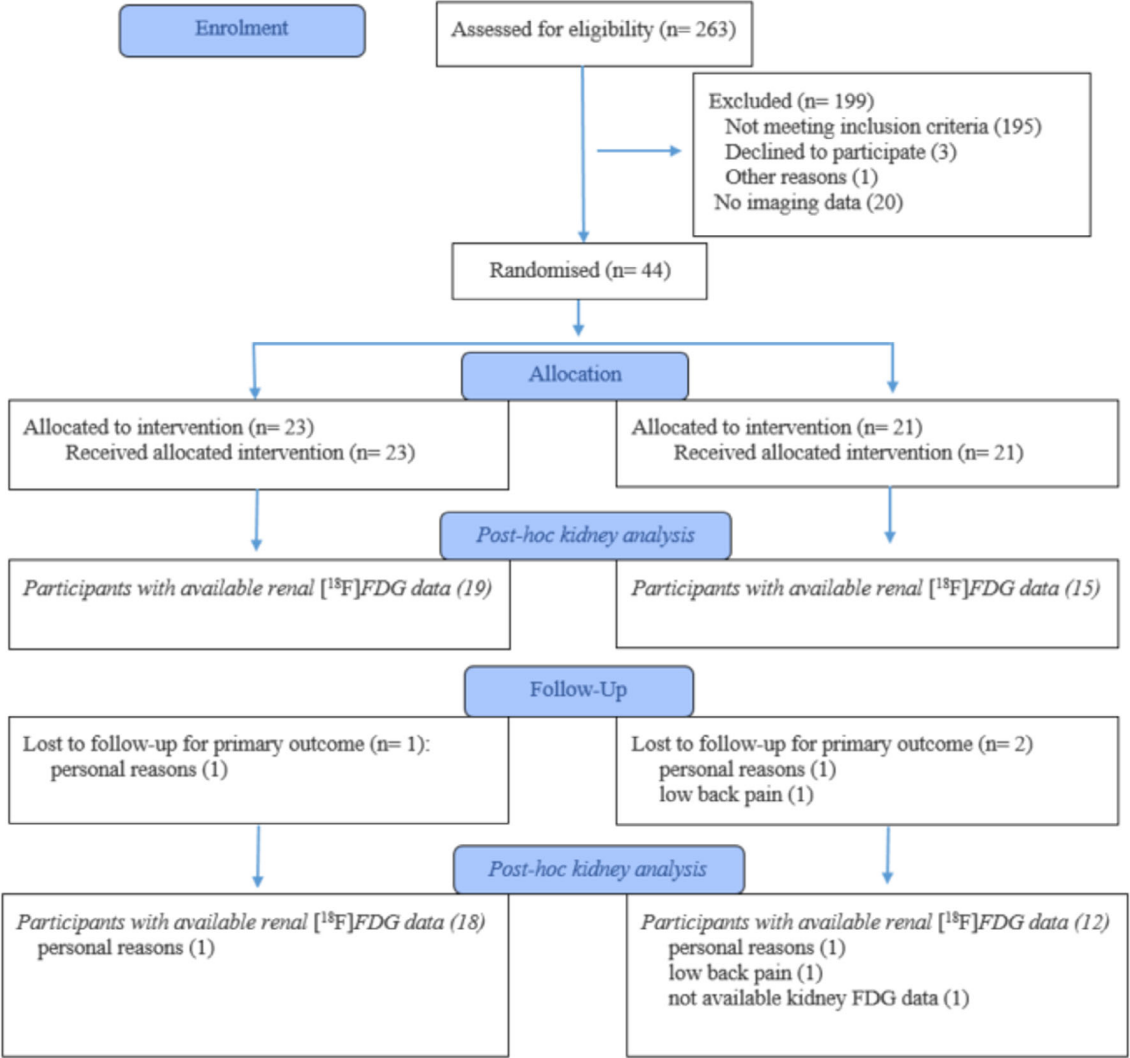
Flow diagram of the progress through the phases of the randomized trial of the two groups including the post‐hoc analysis on the participants who had available [^18^F]FDG kidney data.

Figure 1 shows the flow diagram of the progress through the phases of the randomized trial and the number of participants included in this post‐hoc analysis.

### 2.2 Accelerometry

The methods for the evaluation of accelerometry have been earlier described.^12^ In brief, sedentary behaviour (sitting and lying), standing, light physical activity (PA), and moderate‐to‐vigorous PA were assessed for 4 weeks at the screening phase with a hip‐worn triaxial accelerometer (UKKAM30, UKK Institute, Tampere, Finland). The evaluations were re‐assessed during the whole intervention with a hip‐worn triaxial accelerometer (Movesense, Suunto, Vantaa, Finland) with embedded measurement and analysis algorithms (ExSed, UKK Institute, Tampere, Finland).

Two different accelerometers were used due to their different functionalities and roles in the study. The UKK AM30 is a datalogger‐type accelerometer that passively records physical activity without providing any feedback to participants. It was used to assess baseline physical behaviour. ExSed is an interactive accelerometer that offers real‐time feedback to participants through a mobile application. The intensity of physical activity was measured with the mean amplitude deviation (MAD) in 6‐s epochs. A MAD value of 91 mg corresponded to an intensity of 3.0 METs, while 414 mg represented 6.0 METs.^13^ For detecting sedentary behaviour and postural allocation, epochs with predicted intensity below 1.5 METs (i.e., MAD ≤ 22.5 mg) were further analysed with the angle for postural estimation method (APE). Body posture was classified as standing if the estimated posture angle was less than 11.6°, and as sedentary (sitting or lying) if the angle exceeded this threshold.^13^

### 2.3 Biochemical and anthropometric measurements

After an overnight fast, blood samples were collected and analysed at Turku University Hospital using standard procedures.^11^ Plasma insulin levels were measured by electrochemiluminescence immunoassay (Cobas 8000 e801, Roche Diagnostics). Plasma glucose was assessed using an enzymatic reference method, while plasma triglycerides and total, LDL, and HDL cholesterol were measured with enzymatic colourimetric tests (Cobas 8000 c702, Roche Diagnostics). HbA_1c_ (glycated haemoglobin) was determined by turbidimetric inhibition immunoassay (Cobas 6000 c501, Roche Diagnostics). Body fat percentage, body mass, and fat‐free mass were measured using air displacement plethysmography (Cosmed USA) after at least 4 h of fasting.^11^ Waist circumference was measured at the midpoint between the iliac crest and the lowest rib using a flexible measuring tape. Estimated glomerular filtration rate (eGFR) was calculated using the CKD‐EPI formula.^14^

### 2.4 Hyperinsulinemic‐euglycemic clamp and [ ^18^F] FDG‐PET imaging

In brief, participants were admitted to the Turku PET Centre facilities following an overnight fast. They were instructed to refrain from taking their morning medication, avoid physical activity in the days prior, and minimise their morning step count before arrival. Around 8:00 o'clock in the morning, the hyperinsulinemic‐euglycemic clamp was employed to measure whole‐body insulin sensitivity, as previously described.^15^ A primed‐continuous insulin infusion started with a concomitant variable 20% dextrose solution to maintain plasma glucose levels steady at ~5 mmol/L. During the clamp, glucose was measured in 5–10 min intervals, whereas insulin and free‐fatty acids (FFA) were measured at 0, 80, 115, 135, and 155 min into the clamp. Approximately 60 min into the clamp, participants were positioned inside the PET scanner (GE D690 PET/CT, GE Healthcare, Milwaukee, WI), and 75 (SD 12) min into the clamp [^18^F]FDG was injected [168 (SD 11) MBq] into an antecubital vein and PET imaging started. Blood samples for plasma radioactivity determination (1480 Wizard 3; Wallac, Turku, Finland) and input function calculation were collected for approximately 50 and 70 min after injection, with precise timing recorded. PET image data were corrected for dead time, decay, and photon attenuation and reconstructed using the three‐dimensional ordered subsets expectation–maximisation (3D‐OSEM) method.^12^

### 2.5 Quantification of renal glucose uptake using PET/CT imaging

**FIGURE 2:**
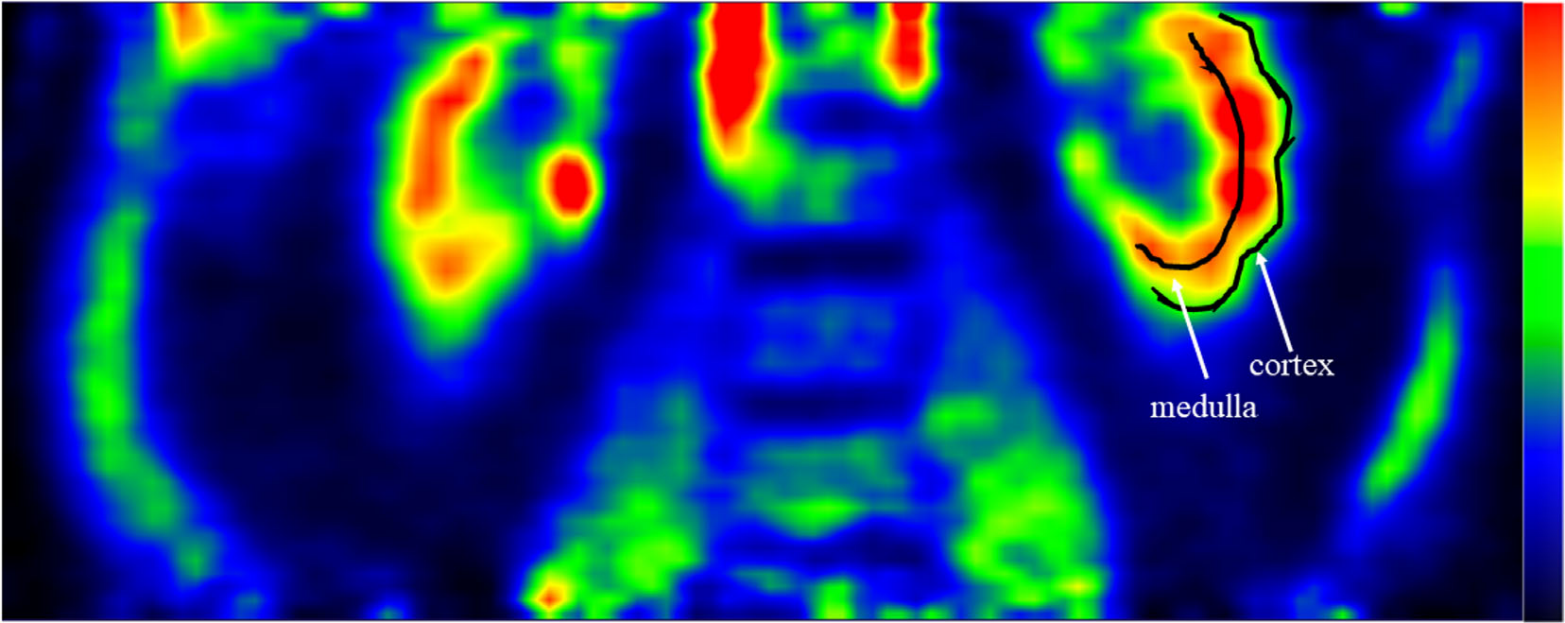
Representative images of region of interest placement in the renal cortex and renal medullary region.

Regions of interest (ROIs) were manually drawn on PET/CT fusion images to obtain time‐radioactivity curves for the renal cortex and medulla (Figure 2). Specifically, 4–5 consecutive thin ROIs were drawn on coronal axis images in the region of slightly lower radioactivity adjacent to the high signal from the renal pyramids, representing the renal cortex. Another thin ROI was drawn centrally on the same slices to represent the medulla. Data were analysed using the fractional uptake rate (FUR, 1/min). Then, corrected FUR values were calculated by subtracting late tubular [^18^F]FDG radioactivity from the ROI activity, as previously described in detail^9^ (Table S1). To determine glucose uptake rates, FUR values were multiplied by the concomitant plasma glucose levels. The lumped constant correction for glucose and [^18^F]FDG uptake in the kidneys was assumed to be 1.^9^ Glucose uptake (GU) rates are expressed as μmol min^−1^ 100 mL^−1^. Liver and skeletal muscle GU rates from this study have been reported earlier^12^, ^16^ and these data were used here to control for associations with renal GU.

### 2.6 Dietary intake

The data regarding dietary intake have been previously published.^17^ In brief, study participants were instructed to maintain their regular dietary habits throughout the study duration. Total daily energy intake was determined through the collection of 4‐day food diaries, which encompassed at least one weekend day. A computerized software (AivoDiet, Aivo, Turku) based on the Finnish Food Composition Database was used to analyse the dietary data.

### 2.7 Statistical analysis

Data are presented as mean (SD) or median [interquartile range], as appropriate. The normality of distribution was assessed using the Shapiro–Wilk test. Baseline comparisons between INT and CONT were done using the *t* test or the Wilcoxon test, as appropriate. Univariate correlations of cortical and medullary GU with clinical parameters and circulating metabolites were presented using Pearson's correlation coefficient and 95% confidence interval for the coefficient. The correlations between the changes in cortical, medullary, and liver GU were presented using Pearson's correlation coefficient and 95% confidence interval for the coefficient among all participants, regardless of the original group allocation. Intervention effects were analysed by linear mixed effect model for repeated measures, with group and time as factors and their interaction. A multivariate linear regression model was constructed to assess the independent associations between cortical radioactivity, late tubular radioactivity, plasma [^18^F]FDG area‐under‐the‐curve and test. A similar multivariate linear regression model was constructed for medullary radioactivity. Standardized β coefficients (St.β) were reported to facilitate the comparison of effect sizes across variables with different units. All statistical analyses were performed on JMP version 17 Pro (SAS Institute, Cary, NC, USA) and SAS software, Version 9.4 of the SAS System for Windows (SAS Institute Inc., Cary, NC, USA). A two‐tailed *p* value <0.05 was considered statistically significant. The sample size calculation was performed based on the primary outcome (change in skeletal muscle insulin sensitivity), as previously described.^12^ However, this was a *post‐hoc* analysis of the trial, and a smaller number of participants had evaluable kidney data as shown in Figure 1.

## 3 RESULTS

Nineteen participants who were randomized to INT and 15 participants randomized to CONT had available baseline data of kidney [^18^F]FDG radioactivity; of them, 18 participants from the INT group and 12 from the CONT group also had 6‐month measurements of renal GU (Figure 1). Characteristics and clinical measurements of the study participants at baseline and at 6 months are shown in Table 1.

**TABLE 1:** Characteristics and clinical measurements of the study participants at baseline and after the intervention.^a^

	CONT		INT		*p*_group_	*p*_time_	*p*_group*time_
	Baseline	6 months	Baseline	6 months			
M/F (*n*)	8/7	6/6	7/12	7/11			
Age (years)	59 [51–61]	—	62 [58–65]	—	**0.04**	—	—
BMI (kg/m^2^)	32.4 [29.2–35.1]	31.7 [28.6–34.2]	32.0 [28.5–36.5]	31.2 [28.3–36.9]	0.7	**0.04**	0.5
Body weight (kg)	93 [85–110]	91 [83–108]	93 [75–103]	91 [75–104]	0.4	**0.04**	0.6
Fat mass (kg)	42.9 (10.8)	41.1 (11.4)	41.4 (10.5)	40.3 (11.0)	0.8	**0.03**	0.5
Waist (cm)	110 [105–125]	108 [103–123]	114 [101–122]	114 [97–119]	0.8	**0.0004**	0.7
M value (μmol min^−1^ kg^−1^)	12.8 [8.2–21.0]	11.4 [9.9–22.4]	15.4 [10.5–21.0]	16.0 [12.3–24.4]	0.8	0.3	0.7
M value (μmol min^−1^ kg_FFM_^−1^)	**21.2 [15.7–28.3]**	**22.5 [16.7–34.9]**	**30.4 [19.8–41.4]**	**28.7 [19.9–45.3]**	**0.7**	**0.4**	**0.6**
eGFR (mL min^−1^ 1.73 m^−2^)	87 [81–95]	74 [60–90]	87 [77–94]	77 [72–86]	>0.9	0.5	0.7
Systolic BP (mmHg)	142 [131–150]	137 [124–141]	151 [136–157]	141 [132–154]	**0.04**	**0.04**	0.4
Diastolic BP (mmHg)	87 [84–98]	85 [77–90]	90 [86–95]	90 [86–94]	0.2	0.05	0.1
Fasting glucose (mmol/L)	5.7 [5.6–6.1]	5.9 [5.6–5.9]	5.8 [5.5–6.0]	5.9 [5.5–6.1]	0.9	0.8	0.5
HbA_1c_ (mmol/mol)	**37 [36–39]**	**38 [36–40]**	**38 [36–40]**	**38 [35–39]**	**0.8**	**0.09**	**0.2**
Total cholesterol (mmol/L)	4.3 [4.1–4.8]	4.6 [4.1–5.0]	4.6 [3.9–5.2]	4.6 [4.0–5.8]	0.8	0.3	0.6
LDL cholesterol (mmol/L)	2.9 [2.6–3.4]	3.2 [2.9–3.4]	3.2 [2.5–3.4]	3.1 [2.5–3.9]	0.2	0.8	0.9
HDL cholesterol (mmol/L)	1.24 [1.03–1.45]	1.17 [1.06–1.45]	1.27 [1.02–1.50]	1.26 [1.06–1.45]	0.9	0.6	0.2
Triglycerides (mmol/L)	1.1 [0.7–1.6]	1.2 [0.9–1.6]	1.4 [0.9–1.6]	1.2 [1–1.8]	0.6	0.3	0.9
ssFFA (mmol/L)	0.09 [0.04–0.19]	0.09 [0.06–0.18]	0.10 [0.05–0.14]	0.07 [0.04–0.13]	0.6	0.6	0.7
Sedentary time (h/day)	10.3 (0.9)	10.5 (0.6)	10.1 (1.1)	9.7 (1.1)	0.06	0.6	0.2
Light PA (h/day)	1.8 (0.5)	2.1 (0.6)	1.7 (0.4)	2.1 (0.4)	0.8	**0.0002**	0.6
Moderate‐to‐vigorous PA (h/day)	0.9 (0.4)	1.1 (0.3)	0.9 (0.3)	1.3 (0.4)	0.2	**<0.0001**	0.07
Number of steps (per day)	4906 (1652)	6553 (1746)	5057 (2055)	8300 (1900)	0.1	**<0.0001**	**0.01**
Energy intake (kcal/day)	1789 [1378–2152]	1828 [1639–2124]	1632 [1469–1968]	1811 [1359–2154]	0.6	0.5	0.4
Liver GU (μmol min^−1^ 100 mL^−1^)	2.6 1.3–3.1]	3.1 [2.3–4.4]	2.5 [1.8–3.3]	3.9 [2.7–7.3]	0.2	**0.002**	0.2
Muscle GU (μmol min^−1^ 100 mL^−1^)	2.1 [1.3–3.7]	2.8 [1.6–4.5]	3.2 [1.5–4.6]	3.2 [2.4–5.0]	0.8	0.7	0.4

*Note*: Bold indicate the signficant differences.

Abbreviations: BP, blood pressure; eGFR, estimated glomerular filtration rate; GU, glucose uptake; PA, physical activity; ssFFA, steady‐state free fatty acid.

^a^ Data are mean (SD), or median [interquartile range].

### 3.1 Baseline renal measurements

Cortical and medullary radioactivity (KBq/mL) were directly related to late tubular radioactivity (Figure S1). This finding lends support to the use of a correction to estimate the tissue GU in both regions, as previously described.^9^ Therefore, cortical and medullary GU refer to the corrected data.

**FIGURE 3:**
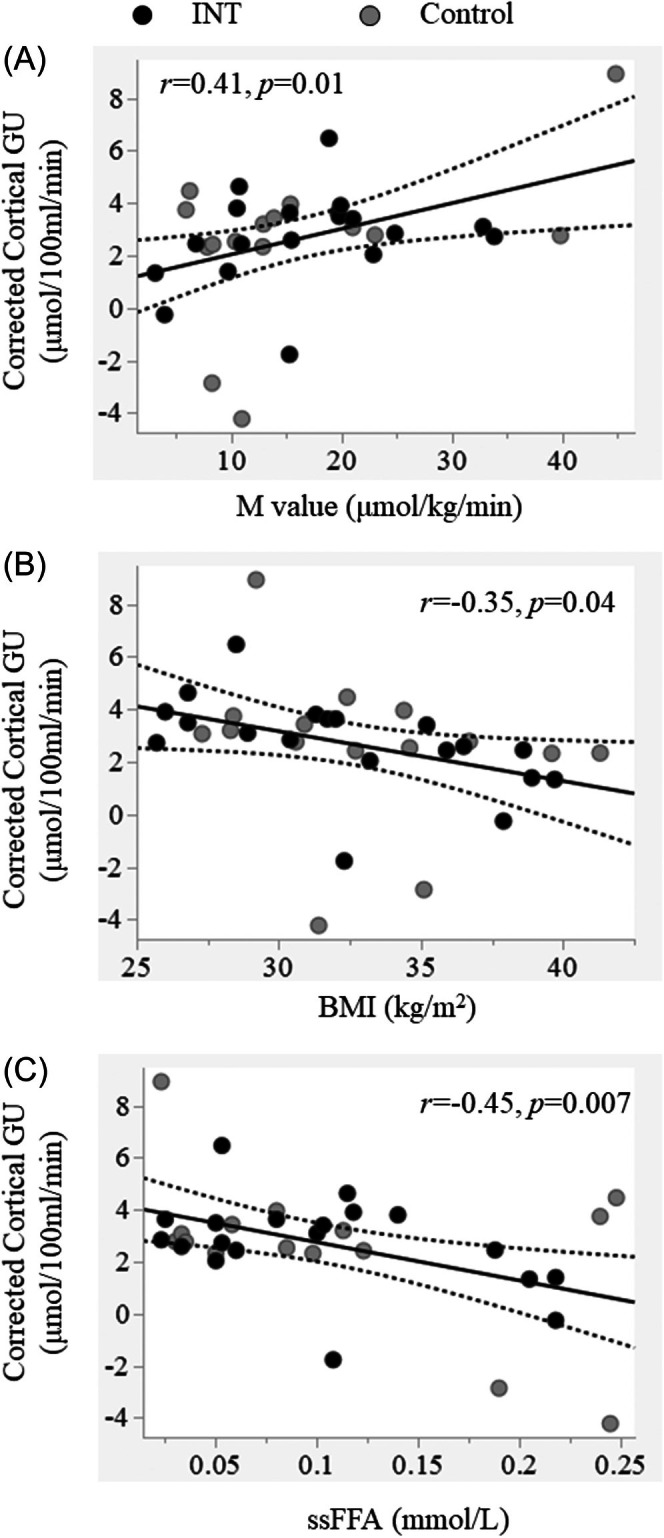
At baseline, cortical GU was directly associated with the degree of insulin sensitivity (M value) (A), inversely associated with BMI (B), and with steady‐state free‐fatty acid levels (ssFFA) measured during the clamp (C). Black circles indicate the intervention group; grey circles indicate the control group.

There were no baseline differences in cortical and medullary GU between the two groups (Table 2). Across all subjects, cortical GU was directly associated with whole‐body insulin sensitivity (M value) (*r* = 0.41, *p* = 0.01) and inversely with BMI (*r* = −0.35, *p* = 0.04) (Figure 3). Cortical GU was also inversely related to circulating steady‐state FFA levels during the clamp (*r* = −0.45, *p* = 0.007) (Figure 3). None of these associations were significant when assessing medullary GU.

**TABLE 2:** Regional renal glucose uptake in the intervention (INT) and control (CON) groups before and after the intervention^a^.

	CON		INT		*p*_group_	*p*_time_	*p*_t*g_
	Baseline	6 months	Baseline	6 months			
Cortical FUR	0.011 [0.009–0.013]	0.011 [0.009–0.013]	0.010 [0.008–0.012]	0.012 [0.011–0.014]	0.8	0.1	0.2
Cortical GU	2.60 [1.40–3.80]	4.10 [2.75–5.45]	2.74 [1.67–3.80]	4.54 [3.45–5.64]	0.6	**0.01**	0.8
Medullary FUR	0.020 [0.016–0.022]	0.019 [0.015–0.024]	0.019 [0.015–0.022]	0.023 [0.019–0.026]	0.6	0.3	0.1
Medullary GU	7.04 [5.44–8.63]	7.97 [6.18–9.76]	7.02 [5.60–8.43]	9.50 [8.04–10.095]	0.3	**0.04**	0.3

*Note*: Bold indicate the signficant differences.

^a^ Data are model‐based means and 95% confidence intervals; Fractional uptake rate (FUR) is in units of 1/min; Glucose uptake (GU) is in units of μmol min^−1^ 100 mL^−1^.

There were no differences in cortical and medullary GU between men and women (Table S2). Moreover, we did not detect any association between renal GU and measures of physical activity (number of steps, sedentary time, light or moderate‐to‐vigorous physical activity per day). The presence of hypertension, or the type of antihypertensive medication, was not associated with renal GU.

### 3.2 6‐month measurements

All study participants, regardless of group allocation, showed an increase in time spent in light and moderate‐to‐vigorous physical activity. Moreover, although all study participants increased the number of daily steps, this increase was more marked in subjects in the INT compared to the CON group (Table 1). Across all study participants, total daily energy intake did not change (Table 1).

After 6 months, participants in both groups had lost weight; waist circumference, fat mass, and urinary clearance of [^18^F]FDG also decreased (Tables 1 and 2). Neither whole‐body glucose disposal nor skeletal muscle GU were significantly different from baseline in either group (Table 1).

Cortical and medullary GU increased significantly in both groups (Table 2).

However, the changes in cortical and medullary GU were not associated with changes in BMI, whole‐body insulin sensitivity (M value), physical activity or with any nutritional variables (data not shown). Interestingly, despite no significant differences in physical activity, sedentary behaviour, weight loss, or insulin sensitivity between men and women, a time‐by‐sex interaction emerged for renal GU. Men showed a significant increase in medullary GU by the end of the intervention, whereas women exhibited comparable medullary GU at baseline and follow‐up. A similar, though non‐significant, trend was observed for cortical GU (Table S2).

### 3.3 Associations between renal, liver and skeletal muscle GU

**FIGURE 4:**
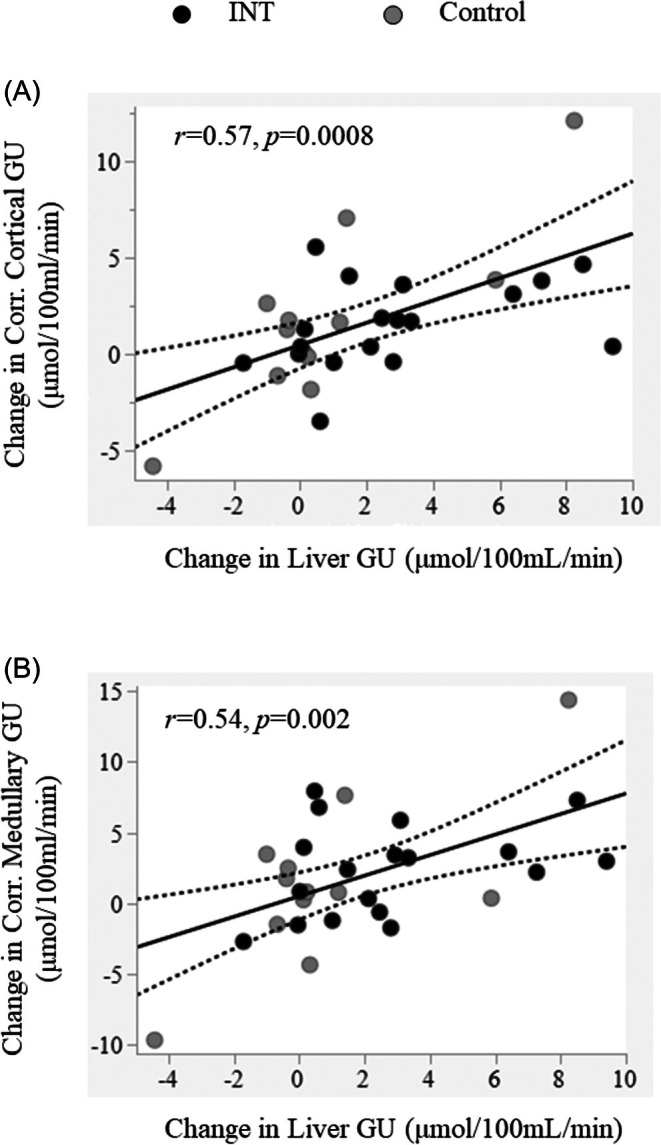
Change in cortical (A) and medullary GU (B) were directly related with change in liver GU. Black circles indicate the intervention group; grey circles indicate the control group.

At baseline, increased cortical GU correlated with higher liver GU (*r* = 0.41, *p* = 0.02), and medullary GU tended to correlate with liver GU (*r* = 0.29, *p* = 0.09). Furthermore, the changes from baseline to 6‐months in both cortical and medullary GU were directly associated with the corresponding change in liver GU among all participants, regardless of original group allocation (*r* = 0.57, *p* = 0.0008 and *r* = 0.54, *p* = 0.002, respectively) (Figure 4).

Cortical GU showed a significant association with skeletal muscle GU at baseline (*r* = 0.53, *p* = 0.001). However, no correlations were found between the respective changes in GU over time.

## 4 DISCUSSION

The main finding of this study was that both cortical and medullary GU increased from baseline to 6 months of intervention among all participants, with no significant treatment effect of the intervention vs control. Indeed, both groups showed similar increases in light and moderate‐to‐vigorous physical activity, with only a statistically larger increase in the total number of steps per day in the intervention group. This enhanced light physical activity, while keeping the same dietary habits, could explain the small weight loss that was observed equally in both groups.

Thus, from a physiological standpoint, the increase in renal GU could be attributed to the increase in light and moderate‐to‐vigorous physical activity and the concomitant decrease in body weight, waist circumference, and fat mass, which occurred in both groups. Although weight loss in the present study was rather small (averaging only 2 kg), it is well established that weight loss in subjects with metabolic syndrome occurs first from the abdominal region.^18^ As we and others have suggested that the renal cortex is an insulin‐sensitive tissue,^9^, ^10^, ^19^ the increase in cortical GU in response to a decrease in visceral adiposity could be regarded as an enhancement of insulin sensitivity in this region. It remains unclear whether the increase in medullary GU is attributable to improved insulin sensitivity or another factor, given that the medulla exhibits markedly different metabolism and perfusion compared to the renal cortex.^20^, ^21^ It is also intriguing that when assessing the effect of sex, a clear time‐by‐sex interaction emerged for medullary GU only, despite similar changes in physical activity, adiposity, and insulin sensitivity in both men and women. Future larger studies should consider this sex effect in order first to confirm it and to address the potential mechanisms involved.

Of interest, a recent systematic review and meta‐analysis incorporating data of 996 489 participants examined the impact of sedentary behaviour on CKD risk. The authors reported that prolonged sedentary behaviour is associated with an increased risk of CKD.^22^ Although our study involved a relatively short follow‐up period of 6 months, it would be valuable to investigate whether renal GU or changes in renal GU predict long‐term renal function.

Along with the enhancement in renal GU, a concomitant, though not statistically significant, decline in estimated glomerular filtration rate was observed—consistent with the well‐established phenomenon of reduced hyperfiltration following weight loss.^23^, ^24^ Taken together, the increase in renal GU and the small reduction in eGFR may represent potential mechanisms by which light physical activity and weight loss confer renal protection. However, long‐term data are lacking, and the interpretation of renal GU assessed via [^18^F]FDG‐PET remains incomplete. Specifically, the identity of the nephron cell types primarily responsible for [^18^F]FDG uptake is still unclear (10), and whether renal glucose metabolism correlates with or predicts future renal function has yet to be determined.

An important methodological note is that the increase in renal GU could be seen only when the corrected data were used. As shown in Figure S1, cortical and medullary radioactivity (kBq/mL) were directly related to late tubular radioactivity. Thus, this finding lends support to the use of a correction to estimate the tissue GU in both regions, as previously described.^9^ Moreover, we found a decrease in the urinary clearance of [^18^F]FDG after intervention in both groups; as urinary clearance of [^18^F]FDG is used in the formula to adjust renal GU values, applying this correction led to significantly higher tissue GU following the intervention. We cannot directly assess the reasons that may explain the decrease in urinary clearance of [^18^F]FDG from the present data; however, a change in renal haemodynamics might play a role. In fact, previous studies employing either bariatric surgery to induce significant weight loss^21^, ^23^ or moderate‐intensity exercise have shown that these stimuli can significantly decrease renal blood flow.^25^ Although in the present dataset we did not have renal perfusion data, and thus this is just a hypothesis, a small decrease in renal perfusion could also explain the numerical decrease in estimated glomerular filtration rate, as the two measures are directly related.^21^

In line with previous reports in younger populations, we found that baseline cortical GU was associated with metabolic measures; in particular, it was negatively associated with BMI and positively with the M value.^10^ Cortical GU was also inversely related to circulating FFA levels during the insulin clamp; this finding is reminiscent of a regional glucose and FFA competition (i.e., Randle‐like substrate cycle).^10^, ^26^ On the contrary, baseline medullary GU was not significantly related to either the M value or plasma ssFFA. This is in line with the results of our two previous studies in adults with obesity and healthy lean controls, which did not show evidence of insulin regulation of medullary GU.^9^, ^10^

Another salient finding of the present investigation is the close correlation between renal and liver GU, but not with skeletal muscle GU. This randomized controlled trial assessed whether a daily reduction of sedentary behaviour by 1 h has an effect on insulin sensitivity. Whereas this relatively mild intervention did not affect whole body (M value) or skeletal muscle insulin sensitivity,^12^ we have recently demonstrated that liver GU was significantly increased.^17^ Here, we found that renal GU was also enhanced and cortical GU was directly associated with liver GU both at baseline and after 6 months of intervention, as was the change in both. The liver and the kidney share common metabolic characteristics. For example, they are organs with very high energy consumption and are responsible for endogenous glucose production in humans.^27^, ^28^ Second, both organs tend to accumulate fat with chronic caloric excess, and recent studies show a close relationship between metabolic dysfunction–associated fatty liver disease and CKD.^29^, ^30^ Functional imaging enables the examination of both organs in a non‐invasive way.^20^, ^31^ Our study would suggest that the hepatic and renal glucose metabolism is tightly connected, but the exact pathways involved are beyond the scope of this study and need to be addressed in the future.

Strengths of the present investigation are the design of a randomized controlled trial and the application of the gold‐standard method for the assessment of insulin sensitivity. Additionally, we measured the participants' sedentary behaviour and physical activity throughout the six‐month intervention, and the intervention was successful in reducing sedentary behaviour by 40 min/day. Moreover, this study further confirms the previously described method for correcting renal GU rates, in order to unmask variations due to residual intratubular [^18^F]FDG activity. Our study also has limitations. First, the water and salt consumption of the study participants the days before the studies and the amount of liquids given during the studies were not standardized, which may affect both [^18^F]FDG excretion in the urine^32^ and kidney function.^33^, ^34^ Second, [^18^F]FDG rather than a glucose tracer was used, with the fate of [^18^F]FDG in the kidney not being thus far completely elucidated. Third, due to an already extensive study design with several visits, oral glucose tolerance tests were not performed; moreover, adipokines like leptin and cytokines were not measured. Finally, as this was not a pre‐specified analysis of the RCT, interpreting the present findings needs caution and should be considered hypothesis generating.

In conclusion, among participants with metabolic syndrome, a small reduction in body weight and increased physical activity leads to enhancement of cortical GU measured under insulin clamp conditions. Cortical GU demonstrated a close association with liver GU, and its reciprocal relationship with circulating FFA resembles a regional, Randle‐like substrate cycle. Further research is required to elucidate the mechanistic basis of these associations.

## AUTHOR CONTRIBUTIONS

**Eleni Rebelos**: Analysed the data and drafted the manuscript. **Prince Dadson**: Drafted the manuscript. **Saara Laine**: Data curation; formal analysis; funding acquisition; investigation; visualization; writing—original draft; writing—review and editing. **Tanja Sjöros**: Conceptualization; data curation; formal analysis; funding acquisition; investigation; writing—review and editing. **Jooa Norha**: Data curation; formal analysis; investigation; writing—review and editing. **Taru Garthwaite**: Data curation; formal analysis; investigation; writing—review and editing. **Eliisa Löyttyniemi**: Formal analysis; supervision; writing—review and editing. **Olli Eskola**: Methodology; writing—review and editing. **Mikko Koivumäki**: Investigation; writing—review and editing. **Henri Vähä‐Ypyä**: Data curation; formal analysis; methodology; writing—review and editing. **Harri Sievänen**: Formal analysis; methodology; writing—review and editing. **Tommi Vasankari**: Conceptualization; writing—review and editing. **Jussi Hirvonen**: Investigation; writing—review and editing. **Kirsi Laitinen**: Conceptualization; writing –review and editing. **Noora Houttu**: Data curation; investigation; writing –review and editing. **Kari K. Kalliokoski**: Conceptualization; writing—review and editing. **Juhani Knuuti**: Conceptualization; writing—review and editing. **Ele Ferrannini** critically revised the text. **Andrea Mari** analysed the renal data and critically revised the manuscript. **Ilkka Heinonen**: Conceptualization; formal analysis; funding acquisition; investigation; project administration; resources; supervision; writing—review and editing. **Ilkka Heinonen** conceived the study design; acquired funding for the study; supervised the whole project; and revised the text. All authors approved the final version of the text.

## FUNDING INFORMATION

The author(s) declare that financial support was received for the research, authorship, and/or publication of this article. The study was financially supported by the Academy of Finland (324243), Instrumentarium Science Foundation (200034), Turku University Foundation (80519), Juho Vainio Foundation (202300322), Hospital District of South‐West Finland (11212), Finnish Diabetes Research Foundation (180021), Finnish Cultural Foundation (190988) and Yrjö Jahnsson Foundation (20227535).

## CONFLICT OF INTEREST STATEMENT

T.S. received a speaker fee from Pihlajalinna Plc, Tampere, Finland, and J.K. received consultancy fees from GE Healthcare and AstraZeneca and speaker fees from GE Healthcare, Bayer, Lundbeck, Boehringer‐Ingelheim, and Merck, outside of the submitted work. The remaining authors declare that the research was conducted in the absence of any commercial or financial relationships that could be construed as a potential conflict of interest.

## PEER REVIEW

The peer review history for this article is available at https://www.webofscience.com/api/gateway/wos/peer‐review/10.1111/dom.16631.

## ETHICS STATEMENT

All procedures performed in studies involving human participants were in accordance with the ethical standards of the institutional and/or national research committee and with the 1964 Helsinki declaration and its later amendments or comparable ethical standards.

## Supporting information


**Data S1.** Supporting Information.

**Table S2.** Characteristics and clinical measurements of the study participants according to sex at baseline and after the intervention.*

## Data Availability

Some or all datasets generated during and/or analysed during the current study are not publicly available but are available from the corresponding author on reasonable request.
