# Supplementary material for: Effects of reducing sedentary behaviour on renal glucose uptake during insulin stimulation: A post‐hoc analysis of a 6‐month randomized controlled trial
Source: Diabetes Obes Metab. 2025 Jul 22;27(10):5772–81. doi: 10.1111/dom.16631 (PMC12409238; doi:10.1111/dom.16631)
Supplement: Supplementary file 1 — Data S1. Supporting Information. [file DOM-27-5772-s002.docx]

**Effects of reducing sedentary behaviour on renal glucose uptake during insulin stimulation: a post-hoc analysis of a six-month randomized controlled trial**

Eleni Rebelos^1,2*^, Prince Dadson^1^, Tanja Sjöros^1^, Saara Laine^1^, Jooa Norha^1^, Taru Garthwaite^1^, Eliisa Löyttyniemi^3^, Olli Eskola^1^, Mikko Koivumäki^1^, Henri Vähä-Ypyä^4^, Harri Sievänen^4^, Tommi Vasankari^4,5^, Jussi Hirvonen^6^, Kirsi Laitinen^7^, Noora Houttu^7^, Kari K. Kalliokoski^1^, Juhani Knuuti^1^, Ele Ferrannini^8^, Andrea Mari^9^, Ilkka Heinonen^1*^

^1^ Turku PET Centre, University of Turku, Åbo Akademi University and Turku University Hospital, Turku, Finland

^2^ Department of Clinical and Experimental Medicine, University of Pisa, Pisa, Italy

^3^ Department of Biostatistics, University of Turku and Turku University Hospital, Turku, Finland

^4^ The UKK Institute for Health Promotion Research, Tampere, Finland

^5^ Faculty of Medicine and Health Technology, Tampere University, Tampere, Finland

^6^ Department of Radiology, University of Turku and Turku University Hospital, Turku, Finland

^7^ Institute of Biomedicine and Nutrition and Food Research Center, University of Turku, Turku, Finland

^8^ CNR Institute of Clinical Physiology, Pisa, Italy

^9^ CNR Institute of Neuroscience, Padua, Italy

Corresponding author: Eleni Rebelos, MD, PhD, email: [eleni.rebelos@utu.fi](mailto:eleni.rebelos@utu.fi) and Ilkka Heinonen, PhD, email: [ilkka.heinonen@utu.fi](mailto:ilkka.heinonen@utu.fi)

**SUPPLEMENTARY MATERIAL**

We have previously described a method based on which cortical and medullary GU are corrected for the residual intratubular amount of [^18^F]FDG (1). The parameters calculated to obtain this correction are presented in **Supplementary Table 1**.

In further support of the correction, cortical and medullary activity (KBq/mL) were directly related with late tubular activity (**Supplementary Figure 1**), suggesting that a correction for residual [^18^F]FDG inside the tubuli would be necessary in order to estimate cortical and medullary GU rates. Pooled baseline and 6-month (post) were used.

**Supplementary Figure 1 –** Correlation between cortical activity (**A**) and medullary activity (**B**) with late tubular activity. Circles are coloured based on individual plasma activity AUC, which was calculated from injection to renal scan time + 20 min.

**A**)

Parameter Estimates :

|  | t value | Pr(>\|t\|) | Std Beta |
| --- | --- | --- | --- |
| (Intercept) | 3.03 | 0.004 | 0 |
| tubular activity | 2.83 | 0.006 | 0.30 |
| AUC plasma | 5.12 | <0.0001 | 0.53 |
| Test (6 months) | 1.94 | 0.06 | 0.20 |

**B**)

Parameter Estimates:

|  | t value | Pr(>\|t\|) | Std Beta |
| --- | --- | --- | --- |
| (Intercept) | 3.22 | 0.002 | 0 |
| tubular activity | 4.26 | <0.0001 | 0.47 |
| AUC plasma | 2.57 | 0.01 | 0.28 |
| Test (6 months) | 1.25 | 0.22 | 0.14 |

**Supplementary Table 1 – Renal parameters**

|  | CON | | INT | | p*_group_* | p*_time_* | p*_t*g_* |
| --- | --- | --- | --- | --- | --- | --- | --- |
|  | Baseline | 6-month | Baseline | 6-month |  |  |  |
| Urine radioactivity (MBq) | 15 [12-16] | 11 [2-13] | 17 [11-23] | 13 [3-18] | 0.4 | 0.0002 | >0.9 |
| [^18^F]FDG dose (MBq) | 166 [162-175] | 170 [160-175] | 169 [162-179] | 168 [160-174] | >0.9 | 0.5 | 0.4 |
| Urine volume (mL) | 295 [215-522] | 318 [199-595] | 363 [272-537] | 40**4** [269-591] | 0.6 | 0.6 | 0.7 |
| Urinary [^18^F]FDG clearance (mL/min) | 35 [29-45] | 28 [7-35] | 34 [29-50] | 30 [7-44] | 0.8 | **0.003** | 0.4 |
| Mean urinary flow (mL/min) | 3.0 [2.2-5.3] | 3.2 [2.1-6.1] | 3.8 [2.7-5.4] | 4.3 [2.7-6.2] | 0.6 | 0.3 | 0.7 |
| Tubular [^18^F]FDG flow (kBq/min) | 152 [121-167] | 115 [35-135] | 175 [120-238] | 126 [30-193] | 0.4 | **0.0004** | >0.9 |
| Mean tubular [^18^F]FDG activity (kBq/mL) | 54 [28-70] | 22 [7-47] | 37 [26-79] | 29 [7-53] | 0.8 | **0.008** | 0.3 |
| Late tubular [^18^F]FDG activity (kBq/mL) | 29 [11-44] | 9 [5-32] | 18 [12-44] | 12 [4-25] | 0.6 | **0.02** | 0.3 |

*data are median [interquartile range].

**References**

1. Rebelos E, Mari A, Oikonen V, Iida H, Nuutila P, Ferrannini E. Evaluation of renal glucose uptake with [(18)F]FDG-PET: Methodological advancements and metabolic outcomes. Metabolism. 2023 Apr;141:155382.
