# Supplementary material for: Effects of reducing sedentary behaviour on renal glucose uptake during insulin stimulation: A post‐hoc analysis of a 6‐month randomized controlled trial
Source: Diabetes Obes Metab. 2025 Jul 22;27(10):5772–81. doi: 10.1111/dom.16631 (PMC12409238; doi:10.1111/dom.16631)
Supplement: Supplementary file 2 — Table S2. Characteristics and clinical measurements of the study participants according to sex at baseline and after the intervention.* [file DOM-27-5772-s001.docx]

**Suppl. Table 2 –Characteristics and clinical measurements of the study participants according to sex at baseline and after the intervention***

|  | | **Men** | | | **Women** | | **p** | | **p** | **p** |
| --- | --- | --- | --- | --- | --- | --- | --- | --- | --- | --- |
|  | **Baseline** | | **6 months** | **Baseline** | | **6 months** | | ***_group_*** | ***_time_*** | ***_group*time_*** |
| INT/CON (n) | 7/8 | | | 7/12 | | | |  |  |  |
| Age (yrs) | 61 [52-64] | | - | 61 [56-64] | | - | |  | - | - |
| BMI (kg/m^2^) | 32.4 [28.3-35.1] | | 31.7 [27.8-34.6] | 31.7 [29.2-36.5] | | 31.5 [29.4-35.4] | | 0.8 | **0.03** | >0.9 |
| Fat mass (kg) | 40.4 (12.5) | | 38.4 (12.6) | 43.4 (8.8) | | 42.5 (9.4) | | 0.4 | **0.02** | 0.3 |
| Waist (cm) | 114 [109-132] | | 112 [107-128] | 107 [102-117] | | 104 [101-116] | | 0.07 | **0.0003** | 0.4 |
| M value (μmol^.^min^-1.^kg^-1^) | 10.5 [6.2-19.8] | | 10.7 [7.7-17.4] | 15.4 [10.9-23.0] | | 18.6 [13.1-30.7] | | 0.8 | 0.3 | 0.7 |
| M value (μmol^.^min^-1.^kg_FFM_^-1^) | 17.9 [10.8-28.6] | | 18.4 [14.5-27.7] | 32.9 [21.0-47.1] | | 33.5 [24.3-56.5] | | 0.7 | 0.4 | 0.6 |
| Sedentary time (h/day) | 10.1 (1.0) | | 10.0 (0.9) | 10.1 (1.1) | | 10.1 (1.1) | | 0.4 | 0.6 | 0.3 |
| Light PA (h/day) | 1.6 (0.6) | | 1.9 (0.6) | 1.9 (0.3) | | 2.2 (0.4) | | 0.1 | **0.0002** | 0.8 |
| Moderate-to-vigorous PA (h/day) | 0.9 (0.4) | | 1.3 (0.4) | 0.9 (0.3) | | 1.2 (0.3) | | 0.6 | **<0.0001** | 0.2 |
| Number of steps (per day) | 4814 (2244) | | 7285 (2222) | 5130 (1548) | | 7681 (1890) | | 0.6 | **<0.0001** | 0.8 |
| Energy intake (kcal/day) | 1839 [1481-2152] | | 1968 [1667-2174] | 1632 [1378-1922] | | 1670 [1392-2043] | | 0.2 | 0.5 | 0.8 |
| Liver GU (µmol^.^min^-1.^100mL^-1^) | 2.0 [1.1-2.8] | | 3.6 [2.3-8.4] | 2.6 [2.1-3.2] | | 3.4 [2.8-5.5] | | 0.8 | **0.0006** | 0.1 |
| Muscle GU µmol^.^min^-1.^100mL^-1^) | 1.9 [1.3-3.7] | | 2.7 [1.3-4.1] | 3.2 [2.1-5.0] | | 3.4 [2.4-5.7] | | 0.1 | 0.6 | 0.6 |
| Cortical GU (µmol^.^min^-1.^100mL^-1^) | 2.7 [2.1-3.6] | | 5.8 [3.5-7.4] | 2.9 [2.4-3.9] | | 3.6 [3.0-4.9] | | 0.9 | **0.005** | 0.06 |
| Medullary GU (µmol^.^min^-1.^100mL^-1^) | 6.6 [5.4-8.3] | | 10.9 [8.1-12.9] | 7.0 [5.4-9.0] | | 6.8 [5.4-9.7] | | 0.2 | **0.005** | **0.02** |

*data are mean (SD), or median [interquartile range]. PA: physical activity; ssFFA: steady-state free fatty acid; GU: Glucose uptake.
